# Impact of Exposure and Illumination on Texture Classification Based on Raw Spectral Filter Array Images

**DOI:** 10.3390/s23125443

**Published:** 2023-06-08

**Authors:** Omar Elezabi, Sebastien Guesney-Bodet, Jean-Baptiste Thomas

**Affiliations:** Colourlab, Department of Computer Science, Norwegian University of Science and Technology (NTNU), 2815 Gjøvik, Norway; sebastien.guesney@hotmail.fr (S.G.-B.); jean.b.thomas@ntnu.no (J.-B.T.)

**Keywords:** texture classification, spectral filter array, convolutional neural networks

## Abstract

Spectral Filter Array cameras provide a fast and portable solution for spectral imaging. Texture classification from images captured with such a camera usually happens after a demosaicing process, which makes the classification performance rely on the quality of the demosaicing. This work investigates texture classification methods applied directly to the raw image. We trained a Convolutional Neural Network and compared its classification performance to the Local Binary Pattern method. The experiment is based on real SFA images of the objects of the HyTexiLa database and not on simulated data as are often used. We also investigate the role of integration time and illumination on the performance of the classification methods. The Convolutional Neural Network outperforms other texture classification methods even with a small amount of training data. Additionally, we demonstrated the model’s ability to adapt and scale for different environmental conditions such as illumination and exposure compared to other methods. In order to explain these results, we analyze the extracted features of our method and show the ability of the model to recognize different shapes, patterns, and marks in different textures.

## 1. Introduction

Texture is usually divided into two categories, tactile and visual textures. Tactile texture refers to the tangible feel you obtain from touching a surface. Visual texture is the impression an observer obtains from perceiving a texture. Visual texture is related to local spatial variations of simple stimuli such as color, orientation, and intensity in an image [1]. This work focuses on visual textures and investigates different methods to classify them.

Texture analysis and classification play a major role in several applications related to material appearance and surface recognition. Examples of successful use of texture can be found in multiple fields such as food science [2], defect detection [3], and medicine [4,5]. In computer graphics, texture is a fundamental feature to allow faithful rendering of material, and it is important to understand how humans perceive texture and how it can be processed, rendered, simulated, or even reproduced [6,7]. Generally, any application that includes texture will benefit from better and more accurate texture classification and analysis algorithms.

Most texture feature extraction approaches focus on the images produced by color cameras, but some research showed the benefits of additional information for texture analysis, such as adding more spectral bands [8]. Studies investigated the effectiveness of using multi- or hyper-spectral imaging devices for texture analysis as they add more information [8,9] but these devices come with additional acquisition complexity and an increase in processing time because of the additional information.

Several imaging techniques allow the capture of spectral images [10]. Spectral image acquisition results from sampling the scene in three axes: spatial, spectral, and time. Most techniques provide a sequential capture of images with the main drawback of having moving parts in the camera and problems when the camera or the object is moving.

To solve this problem, Spectral Filter Array (SFA) [11] was introduced. This technology uses the same concept as the Color Filter Array (CFA) that is commonly used in color cameras. Similar to CFA, SFA is a single-sensor camera that has different spectrally selective filters in front of each pixel to measure specific bands of the electromagnetic spectrum. The number of bands depends on the pattern used for the SFA. The pattern design is very important since it impacts the full-resolution image reconstruction and defines how selective the camera is. Examples of patterns can be found in the literature, e.g., [12,13,14], and which kind of pattern is the best one is still an open question.

The estimation of the missing bands for each pixel is called demosaicing. Indeed, the SFA sacrifices spatial resolution to gain spectral and temporal advantages. The spatial accuracy thus depends strongly on the demosaicing process. Demosaicing can be considered as a specific case for interpolation, and a huge body of literature investigates how to conduct spatial resolution reconstruction. This work focuses on performing texture classification from raw SFA images without performing demosaicing. This allows for a faster and simpler method and will have the advantage of utilizing the correlation between the different bands without more computation. This is relevant since the demosaicing process does not add additional information to the data captured. Additionally, in this work, we focus on testing the effectiveness of the deep learning/convolutional neural network (CNN) based methods for SFA texture classification directly on the raw image and how it generalizes with the variation of illumination conditions, and exposure time and how it compares with the classical approaches that are based on LBP method.

In the next Section 2, we will describe the related work of texture classification and some of the research done in this area. In Section 3, we will describe the datasets and methods used during our experimentation setup. Finally, in Section 4, we will represent the results we obtained and the conclusions we perceived from the conducted experiments.

## 2. Related Work

During the initial era of digital image processing, most of the literature proposed to compute texture features on a single-channel image, such as a luminance channel. With the generalization of the use of color images, researchers showed that the texture features quality extracted from luminance was improved by considering the color channels and the distribution of pixel values in a color space in addition to the spatial arrangement of the colors in the image plane [15]. This method increased the performance and the quality of the feature extracted and also increased the computational time and the number of characteristic features. Most methods today utilize color images, so new methods were created, and former methods like Local Binary Pattern (LBP) were extended to color images. Additionally, different varieties of LBP were introduced that are more robust to illumination change and noise [16]. The success of learning-based methods, especially deep learning and CNN, on different computer vision tasks, such as image classification or enhancement, encouraged researchers to adapt them to more vision and perception tasks, including texture analysis and classification [17]. One current problem with learning-based methods is the availability of the texture dataset and the performance of the model, which is directly connected to the quality of the dataset and the varieties of textures included. More effort was investigated to see the effect of having extended spectral information on texture analysis. Khan et al. [9] created a high resolution in the spectral and the spatial domain for texture data to study texture analysis utilizing a hyper-spectral imaging device. They also analyzed the effect of using a higher number of bands and showed the importance of the methods that use the correlation between different bands and not only depend on the spatial information of the data. Similarly, Conni et al. [8] studied the effect of the number of bands on the performance of different texture analysis methods. With the performance enhancement from using more bands in texture analysis and classification, more methods were developed, and other methods were adjusted to be used with hyperspectral and multispectral texture data such as k-band LBP [9], CNN [18], and Co-occurrence matrix [19].

Methods that depend on color images generally depend on the demosaiced image. The demosaicing process was designed to produce a standardized color image format for storage, communication, and visualization purposes, so they often use filtering to avoid visible color artifacts, and many of the efficient methods rely on the frequency domain. These methods tend to alter the local texture information of the image, which reduces the texture information we can obtain from the image. Losson et al. [20] investigated this problem and proposed an LPB variant method that works directly on the CFA. They showed that this approach produces better results with less complexity, so they demonstrated that applying texture classification directly on the raw CFA image was a relevant approach. However, this comes with a lack of generalization since the algorithm needs to be adapted to each mosaic pattern. Mihoubi et al. [21] extended this concept to spectral and developed an LBP method that works directly on SFA. Their experiments showed that the method works better than most of the methods that require demosaicing with a much better computational time.

Very recently, Amziane et al. [22] proposed a CNN-based network for texture classification and analysis. Their model consists of three different CNN blocks with max pooling in between and a fully connected network at the end for texture classification. Their model works directly with the MSFA array without demosaicing. Their experimentation is based on a simulated dataset using the HyTexiLa dataset [9] by simulating two different SFA patterns without any variation in illumination and without capturing artifacts or environmental effects. This work was published after we finalized our experimental work, so we have not taken it into account while designing our research.

Our work also investigates the performance of a CNN architecture for texture classification on the same texture data. At the inverse of Amziane et al. [22], we do not perform simulation, and we collected our own SFA dataset with an actual sensor, SILIOS CMS-C [23] under five different illuminations and three different exposure times. In addition, our dataset includes capturing artifacts effects that can be noticed during real applications, such as lens distortion, out-of-focus regions, and vignetting. The addition of different illumination and exposures allowed us to investigate the robustness of our CNN-based model to different illuminations and brightness and showed the ability of our method to work on different illuminations and exposures than the training data. We did not include the results from Amziane et al. [22] as the dataset used is different–simulated data–and the code for their method is not available to train it on our SFA dataset. However, hopefully, the release of our dataset will encourage the use of a real SFA dataset for texture classification methods and will create a new benchmark that will make it possible to compare between different methods developed and will give a better insight into how the methods will behave in actual capturing environments. Additionally, we visualize the saliency map of our model to show what parts of the texture the model focuses on to make the classification decision and showed that our method could differentiate between the different patterns and attributes of different textures.

## 3. Methodology

### 3.1. Dataset

**Figure 1:**
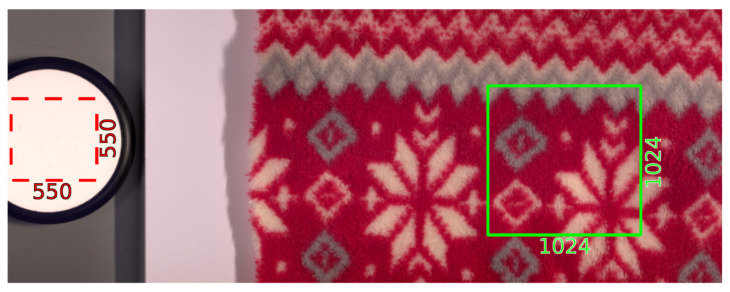
Data acquisition setup of HyTexiLa Dataset. Reproduced without modification from [9].

Our experimentation is based on two different texture image datasets of the same materials. The first dataset is HyTexiLa [9]. This dataset is a high-resolution hyper-spectral dataset for different textured materials from 5 different categories. The dataset was collected using HySpex VNIR-1800 [24], a line scanner hyper-spectral imaging device. The dataset consists of 112 textured materials, 65 of which are textile-textured materials. The spatial resolution of the dataset is 1024 by 1024 with a spectral resolution of 186 bands from the range [400–1000] nm, so the dataset is very high resolution in both spectral and spatial dimensions. The dataset is collected from closeup texture samples, so it is very detailed. The captured data were transformed to reflectance data so they are illumination invariant. For each texture sample, only one capture was taken. An image from the acquisition setup is shown in Figure 1. For this dataset, we only focused on the textile textures materials.

**Figure 2:**
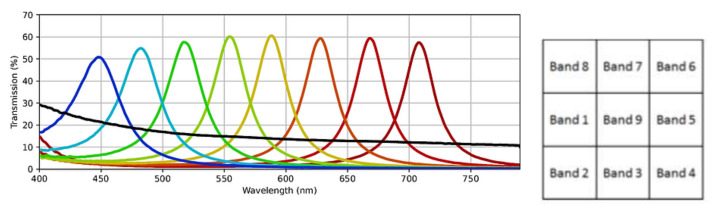
Spectral bands and the SFA Pattern for SILIOS CMS-C [23] sensor. Reproduced without modification from [23]. Numbers of band increase with the wavelength peak, Band 9 being the achromatic band.

**Figure 3:**
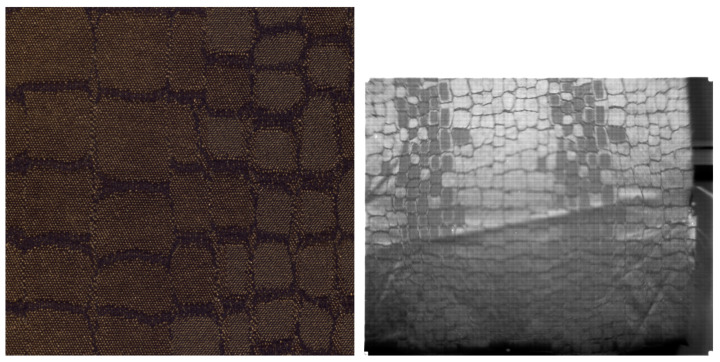
Same textile texture from HyTexiLa dataset (on the **left**) and SILIOS CMS-C dataset (on the **right**).

The second dataset is a new dataset of the same textile materials captured in the HyTexiLa dataset we collected using the (SFA) SILIOS CMS-C [23]. The sensor used has a spatial resolution of 1280 by 1024 and captures nine spectral bands from the range [430–700] nm. The SFA pattern and the spectral bands of the SILIOS CMS-C sensor are shown in Figure 2. This dataset is much less detailed than the first and corresponds to what can really be acquired in the field by today’s technologies. The dataset was captured under five different lighting conditions within a viewing booth (An illuminant, cool white, daylight, horizon, and TL84) and with three different exposure times (20, 50, and 70 ms) for each lighting condition which resulted in 15 different captures for each texture sample. For this dataset, we only have one image capture for each texture under a specific lighting condition and exposure time; additionally, the reader will notice parts of the image that are not sharp or in focus, noise, and other capturing artifacts that can happen during real-world capturing. In addition, the texture images are not at a similar scale to the HyTexiLa dataset (the HyTexiLa dataset capture is zoomed in, but the new dataset has a much wider field of view). Differences between the two dataset images are shown in Figure 3. The data will be made available as Supplementary Materials. In addition, the texture images are not at a similar scale to the HyTexiLa dataset (the HyTexiLa dataset capture is zoomed in, but the new dataset has a much wider field of view). Differences between the two dataset images are shown in Figure 3. The data will be made available as Supplementary Materials.

For both datasets under specific capturing conditions, we only have one capture for each texture class, so in order to use the datasets for training and evaluation, we needed to split each capture into small patches. This choice resulted in having 25 image patches for each texture class under specific capturing conditions, and in the case of the new dataset, if we included all the lighting conditions and exposure times, we would have 375 image patches. This dataset size is very small, especially for deep learning-based methods, in order for them to generalize. We later show how we tried to overcome this issue and how the deep learning-based methods performed with this small amount of data.

### 3.2. Hyper-Spectral Setup

We first test the performance of texture classification in *ideal* conditions on the HyTexiLa data. This performance will set our desired goal when working with real SFA data. The ideal algorithm would give the same performance, as in this ideal case, in different environmental conditions. In the HyTexiLa paper, the authors tested different LBP algorithms that were extended to hyperspectral LBP. These can be applied to the k-number of channels in spectral images. They also tested the effect of the number of bands on the performance of these algorithms. Their experimentation showed that The Opponent Band LBP (OBLBP) was the best-performing algorithm, and the performance stabilized after 10 bands. We followed their experimental setup for their best-performing model, and we recreated their results.

For the preparation of the dataset, we selected 10 bands from the 186 bands of the HyTexiLa. The bands we selected are equally spaced and cover the full spectral range [400–1000] nm. For each texture material, we split its image of size 1024 × 1024 × 10 (number of bands we selected) into 25 non-overlapping batches of size 204 × 204 × 10. Twelve of these batches were randomly selected for training, and the other 13 were used as test images. The OBLBP [25] algorithm was extended to work with k-channels. In the original LBP operator, the LBP operator is applied on each channel separately by only comparing a central pixel to its neighboring from the same channel, and for each channel, you obtain a feature vector and combine all these feature vectors to obtain the final features of size K×size_of_histogram. For OBLBP, one considers the inter-channel correlation by applying the LBP operator for each channel pair. So, instead of comparing a central pixel to its neighboring pixel from the same channel, you compare it to its neighboring pixels from another channel, which result in a feature vector of size $K^{2}\times size\_of\_histogram$. The mathematical formulation of the extended method is shown in Equation (1).

(1) $$OBLBP^{\left(k,l\right)}\left[I\right]\left(p\right)=\sum_{q\in N^{P,d}}s\left(I_{q}^{l},I_{p}^{k}\right).2^{\epsilon (q)}$$

where is $OBLBP^{\left(k,l\right)}$ represents LBP feature between channel *k* and *l* and pixel *p*. $I_{p}^{k}$ represent central pixel in channel k and $I_{q}^{l}$ represent neighboring pixels in channel l. Finally *s*, in Equation (2), represents the comparison operation.

(2) $$s\left(\alpha ,\beta \right)=\left\{\begin{array}{cc} 1 & \mathrm{if}\;\alpha \geq \beta \\ 0 & \mathrm{otherwise} \end{array}\right.$$

For classification, we used a 1-Nearest Neighbor decision algorithm. It works by testing the similarity between each test batch’s features to the features of the batches in the training set using the intersection between histograms algorithm. For each test batch, the most similar batch from the training batches will be the same texture as the test batch.

### 3.3. SFA Setup

In this setup, we classify the SFA dataset. We used a similar setup as the hyper-spectral data, with some modifications to account for the dataset differences. We work with raw images, so the depth of the image we will use is 1, and the spectral information is included in the pattern of the SFA. Because the field of view of the capture in the SFA data is larger, some parts of the texture images do not include texture, as we can notice in Figure 3. We took the middle part of the image of size 510 × 510 as the texture image that we will work with. For this texture image, we split it into 25 non-overlapping batches of size 102 × 102. Twelve of these batches were randomly selected for training, and the other 13 were used as test images. Even though the size of the batches in the SFA data is smaller than the batches in the HyTexiLa data, the field of view in the SFA data will be larger since the field of view in the SFA texture image is much larger than the HyTexiLa texture image field of view.

For the algorithms used for texture features extractor and classification, we tested two different algorithms. The first algorithm is proposed by Mihoubi et al. [21], which is a modified LBP algorithm that works directly on the SFA raw image. The second method is the one proposed by us, which is a learning-based method that utilizes CNN to extract the texture features. The two methods are described next in detail.

#### 3.3.1. LBP SFA

**Figure 4:**
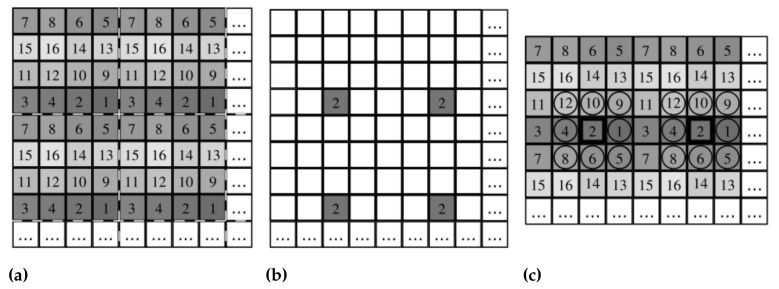
SFA LBP calculation process. (**a**) represent SFA raw image. To calculate the histogram for band 2 we choose the pixel subset $S^{2}$ as we see in (**b**). Then for each pixel in this subset, we calculated the LBP value by comparing it to the neighboring pixels with different bands, as we see in (**c**). Figures from [21].

Mihoubi et al. [21] developed an LBP-based method that works directly with the raw SFA images. This method is analogous to the OBLBP method that was used with the HyTexiLa dataset. This algorithm works by creating a separate LBP histogram for each different band in the SFA, so for SFA with *K* bands, we will have *K* histograms. To create the histogram from the $k^{th}$ band, we select the pixels with this band $S^{k}$ to compute this histogram. For these pixels to compute the LBP value, we compare these pixels to the neighboring pixels from different bands. By comparing these pixels to the neighboring pixels from different bands, we also consider the inter-correlation between the different bands in the SFA capture. The concatenation of all the *K* histograms represents the final texture features of size K×size_of_histogram. The calculation steps are shown in Figure 4. This algorithm was named MSFA-based LBP (MLBP) and follows Equation (3).

(3) $$MLBP\left[I^{raw}\right]\left(p\right)=\sum_{q\in N_{p}}s\left(I_{q}^{l},I_{p}^{k}\right).2^{\epsilon (q)}$$

where $MLBPI^{raw}\left(p\right)$ is the LBP value calculated for pixel *p*. $N_{p}$ are the neighbors of the pixel *p* from different bands. Histogram calculations are described in Equation (4).

(4) $$h^{k}\left[MLBP\left[I^{raw}\right]\right]:\left[0,2^{P}-1\right]\to \left\{0,...,\right|S^{k}\left|\right\}$$

(5) $$j\to |\{p\in S^{k},MLBPI^{raw}\left(p\right)=j\}|$$

where $h^{k}\left[MLBP\left[I^{raw}\right]\right]$ is the histogram of band *k* calculated from $S^{k}$ pixel subset.

For classification, we used 1-Nearest Neighbor, similar to the model we used for the HyTexiLa Dataset.

#### 3.3.2. NN SFA

For our proposed method, we use CNNs to develop a learning-based method based on VGG-11 [26] architecture. We adapted the architecture to our problem by modifying the first layers to work with 1-channel raw images and decreasing the size and shape of the fully connected layers to only one layer to decrease the model size. We choose VGG-11 as it is simple and relatively small in size compared to other CNN architectures commonly used. The smaller size will be beneficial in our case because of the small amount of training data we have. We considered the raw SFA image as a grayscale image. The task formulation is described in Equations (6) and (7).

(6) $$\hat{y}=F\left(I;\theta \right)$$

(7) $$\hat{\theta }=argmin_{\theta }L\left(\hat{y},y\right)$$

where *F* represents the model used for the classification task. $\hat{y}$ represents the prediction of the model. $\hat{\theta }$ represents the optimal parameters for the model, and we can obtain them by choosing the parameters that minimize the error between the model prediction and the ground truth. *L* represents the loss function that we use to calculate the model error and optimize its performance.

The texture dataset we have is very small since we only have 25 image patches for each texture. Deep learning models require a large amount of data to produce a robust model with good performance. To overcome this problem, we used the VGG-11 model trained on ImageNet [27] dataset as our initial weights (pre-trained model). So instead of training the model from the beginning, the new model will utilize the weights that were generated to extract features from the ImageNet dataset and adapt it to work with the texture dataset. This is very helpful when working with a small dataset similar to our case. Even though the pre-trained weights on ImageNet are for a very different task and the images are very different domains, so the leaned features for the two tasks will be very different, we found that starting from this pre-trained model stabilizes the model training and decreases the number of epochs needed for training. We also utilized data augmentation by random rotation, flipping, and brightness to the input patch. We then trained the model for the texture dataset and compared it to the performance of the LBP SFA algorithms and the performance of the OBLBP on the HyTexiLa dataset.

### 3.4. Evaluation Metric

To evaluate the performance of the different algorithms on texture classification, we used F1-score metric. The F1-score calculation depends on the computation of precision and recall. Precision (Equation (8)) represents how the model prediction is precious by calculating all the positive predictions that the model made and how many of them were correct. Recall (Equation (9)) represents the model’s ability for correct prediction by representing from all the positive samples in the test set how many of them the model predicted correctly. F1-score (Equation (10)) is then calculated as the harmonic mean of the precision and the recall.

(8) $$Precision=\frac{TruePostive}{TruePostive+FalsePostive}$$

(9) $$Recall=\frac{TruePostive}{TruePostive+FalseNegative}$$

(10) $$F1-score=\frac{2*Precision*Recall}{Precision+Recall}$$

To calculate the F1-score for a classification task with multiple classes, we first calculate F1-score for each class separately and average the F1-score for all the classes to obtain the model F1-score. To calculate each class F1-score separately, we consider all test samples for this class as positive samples and the rest of the test samples to be negative samples.

We also produce the confusion matrix [28] to visualize the model decision and see, for each texture, what are the other textures that the model confuses this texture with. Because of the large size of the confusion matrix, it will be only included in the report when necessary, but the confusion matrix for all the experiments was analyzed, and the important conclusions from this analysis are mentioned for each experiment.

## 4. Results

### 4.1. HyTexiLa Dataset

Texture classification results on the HyTexiLa Dataset are shown in Table 1. The model performance on the textile textures is 100%. Additionally, for the performance on all textures in the dataset, we obtained a performance of 98%, which matches the performance that was reported in the HyTexiLa paper [9]. These results show us that under ideal conditions, for our small number of textures, without any environmental effects, such as different illuminations or intensities, and with high-resolution, detailed images, the best performance possible on the textile dataset is 100%, which indicates no miss-classified samples.

### 4.2. SFA Dataset

Here, we compare the performances of the different algorithms dedicated to SFA images. During the experiments, we will refer to the SFA-LBP method as LBP SFA and the CNN texture classification method as NN SFA.

#### 4.2.1. Classification on SFA with Similar Acquisition Parameters

For this experiment, we choose one lighting condition and one exposure for both training and testing (12 batches for training and 13 batches for testing). The purpose of this experiment is to test the performance of the SFA algorithms without any environmental changes between the training and testing. We choose daylight lighting conditions with 50 ms exposure time.

As we see from the results in Table 2, the performance of our proposed method (NN) is better than the LBP-based method. Even with the small amount of data in the texture dataset, the learning-based method was able to learn the different patterns in the different textures and was able to outperform the LBP method. Additionally, we noticed in the confusion matrix of both methods that the NN model struggled less with the classification of textures and had fewer false detection than the other LBP model.

The method with the best performance was still less performant (97% vs. 100%) than the texture classification performance with the HyTexiLa dataset, which shows the effect of environmental conditions, capturing artifacts, and the data quality on the model performance.

#### 4.2.2. Algorithm Robustness against Different Illuminations

To test the performance of the model robustness under different lighting conditions, we conducted multiple experiments. For the first one, we included all the textures captured under different lighting conditions for both training and testing, see Table 3) to see the effect of adding more environmental conditions to the dataset. In the second experiment, we used all the batches of texture images under a certain lighting condition for training (horizon illumination) and the same textures under different lighting conditions for testing (daylight illumination), see Table 4 to see how the methods generalize to unseen environmental conditions. For the third experiment, we used one lighting condition for testing (daylight illumination) and the rest of the lighting for training, see Table 5, to see how the models adapt to the unseen environmental conditions when we increase the variety of environmental conditions in the training set. For all these experiments, we used images with an exposure time of 50 ms.

From all these results, we observe that the model performance dramatically decreases when introducing different lighting conditions. This is because different lighting conditions affect different bands differently, so the spatial difference between different bands changes for different lighting conditions. This makes texture features extracted very different under different lighting conditions. This will make the classification task harder for the methods, which will decrease the performance, as we notice in Table 3.

**Figure 5:**
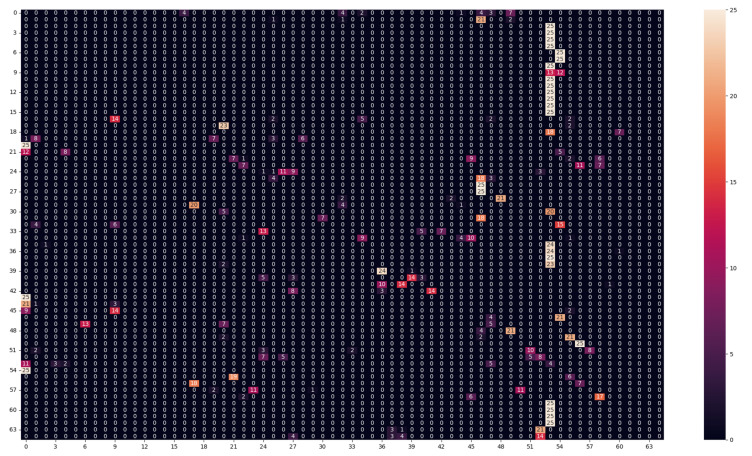
Confusion matrix of the LBP model in (Daylight for testing–horizon for training–50 ms) experiment.

**Figure 6:**
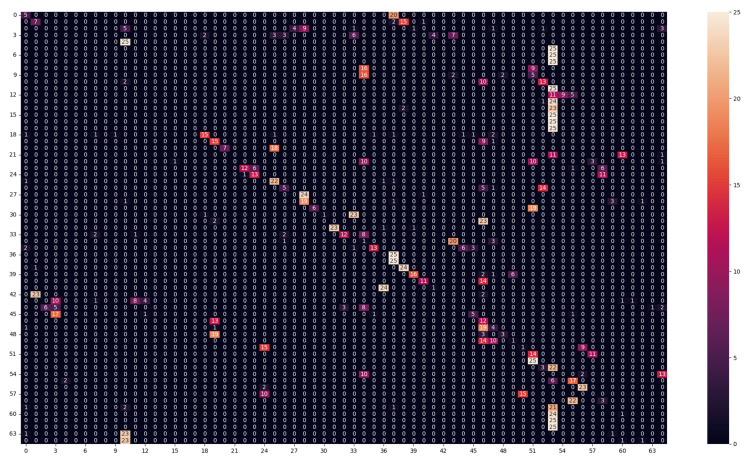
Confusion matrix of the NN model in (Daylight for testing–horizon for training–50 ms) experiment.

As we notice in Table 4, the model performance suffered severely. Both models depended on the spatial distribution of the bands under the training lighting conditions, as changing lighting conditions will affect the different bands differently, so the relative values between the different bands will change. Because of the nature of the LBP method, the features are calculated by the difference between a center pixel with a specific band and the neighboring pixels with different bands. Because changing the lighting conditions changes the relative values between the different bands, the features of the same texture under different lighting conditions will be very different. Similarly, the CNN-based method was also dependent on the relative values between the different bands; the textures under different lighting conditions appeared totally different for the classification algorithms, which resulted in the drop in performance of both methods. The model performance was most likely trying to classify textures that it had never seen before. Additionally, from the analysis of the confusion matrix for both models (Figure 5 and Figure 6), the most false-negative classes were classes 53 and 46. These classes were for textures with blue colors (false negatives), as we noticed in Class 46 - textile_20_blue and Class 53 - textile_22_blue. This can be explained because the test lighting conditions (daylight-D65) have more power in the blue region than the training lighting conditions (horizon-D50), so most of the textures will appear bluer to the models because the bands in the blue regions will have more energy than the textures under (D50). So the models predicted different textures as textures with blue colors.

As mentioned before, the purpose of the third experiment was to see whether adding more lighting conditions during training would help the models to adapt better to new lighting conditions. As we notice in Table 5, the performance of the LBP model is kept very low since the LBP process is fixed and depends on the difference between the neighboring pixel to extract the features, so it did not adapt well to new lighting conditions. Conversely, the NN model performance increased since the feature extraction process changed with the change in the training data, as the feature extraction process is learnable and changes based on the training data. We can notice the performance increase in the confusion matrix of the model, and the model decision is not fixed for blue textures as in the previous experiment. The NN model performance is still far from the desired performance, but this experiment showed the ability of the model to adapt to different lighting conditions with the increase in the data variety during the training process. Thus the addition of more representative data with a variety of lighting conditions would make the model more robust for the different lighting conditions.

#### 4.2.3. Algorithm Robustness against Different Exposures

We repeated the same experiments we performed in the previous part, but we varied the exposure instead of the lighting condition. These experiments were used to test the robustness of the models for images captured with different exposure, i.e., of varying light intensities. For the first experiment, we included all the textures captured with different exposure times for both training and testing. In the second experiment, we used all the batches of texture images captured with a certain exposure time for training (20 ms) and other batches of texture images captured with another exposure time for testing (50 ms). For the third experiment, we used one exposure time for testing (50 ms) and the rest of the exposure times for training. For all these experiments, we used images captured under daylight illumination.

Testing the models for different exposure times can be considered as simulating different lighting intensities. Changing exposure time does not have as big an effect as changing the lighting conditions since the bands are affected equally, so the relative difference between the different bands in the SFA raw image will not change much. In the first experiment, as we notice in Table 6, adding new exposures for both training and evaluations increased the performance of both models. Because changing the exposure did not change the difference between the different bands adding new exposures was like adding more data for the same textures, which helped both models to achieve better performance.

For the second experiment, when we had only one exposure for training and a different one for evaluations, we noticed a performance drop in both algorithms, as shown in Table 7. The LBP model performed better, as the feature extraction process for LBP depends on the relative difference between the different bands, which is not heavily affected by exposure time. However, the performance of the CNN model was very low. This suggests that the model learned amplitude-based features, which are heavily affected by the exposure time, or that the noise was predominant for such a short exposure time.

The third experiment result is shown in Table 8. When we introduced different exposure times for the training data, both models adapted to the difference that happened because of the change in exposure, and both performed very well, especially the CNN-based method, which again showed the ability of the method to generalize better by increasing variety in the training dataset. These experiments showed that adding more data with a variety of exposure times helps the model to achieve better performance and helps the models to be more robust against the change in illumination intensities.

In our experiments, all the exposure we had did not result in sensor saturation, so we did not have overexposed images. It will be interesting to investigate how the different models will perform under such constraints.

### 4.3. Neural Network Model Visualizations

To investigate what kind of features the neural network model focuses on to perform the classification decision, we used a saliency map. Saliency maps show what parts of the input image the neural network considers important features for a given texture. This helps us to visualize what the CNN architecture did learn and what kind of features it tries to extract. We used a saliency map variant called Grad-cam [29] in our experiment.

**Figure 7:**
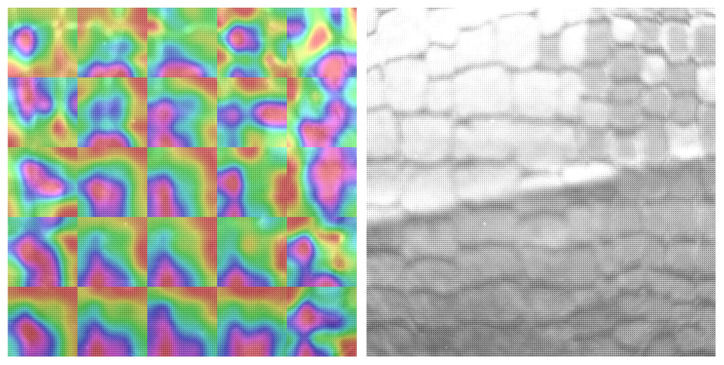
SFA texture image on the right, Saliency map laid on the SFA image on the left, textile_01_back daylight 50 ms.

**Figure 8:**
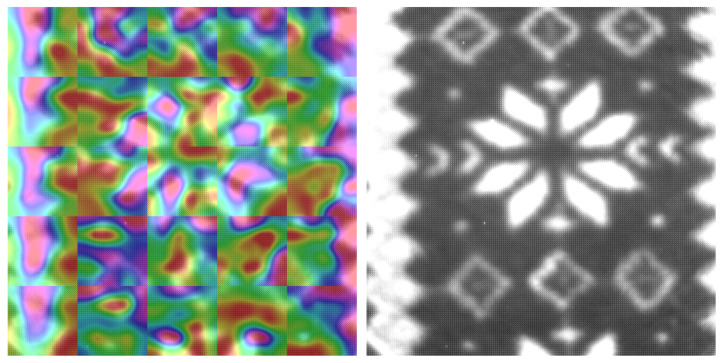
SFA texture image on the right, Saliency map laid on the SFA image on the left, textile_13_blue daylight 50 ms.

**Figure 9:**
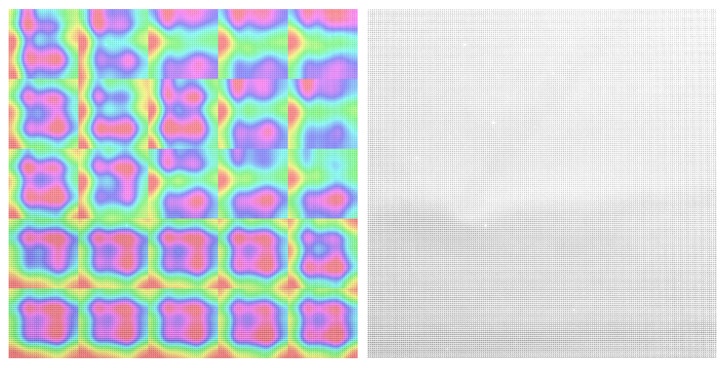
SFA texture image on the right, Saliency map laid on the SFA image on the left, textile_14_red daylight 50 ms.

**Figure 10:**
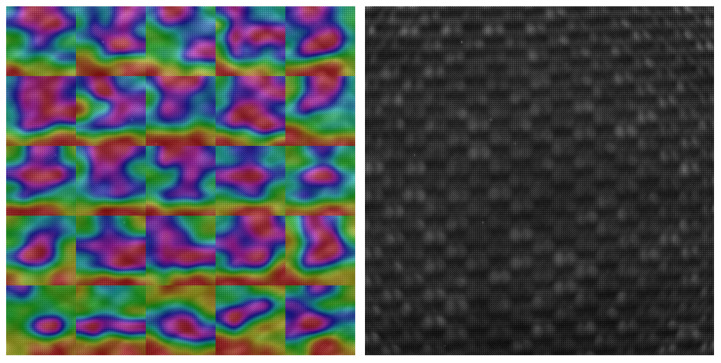
SFA texture image on the right, Saliency map laid on the SFA image on the left, textile_18_green daylight 50 ms.

We visualized the saliency maps of different textures with different patterns and different unique marks. As we see in all visualizations in Figure 7, Figure 8, Figure 9 and Figure 10, we can notice the models focusing on the unique features of each texture.

In Figure 7, we notice the model focuses on the unique lines in the texture. As we see from the saliency map, the parts of the image that have the leather lines are the most important to the classification decision, which shows that CNN learns the unique features of each texture. We can also notice the model adapts for each area in the texture image and is not focusing on a specific area.

In Figure 8, we can notice that the texture has unique shapes in the center and at the edges. The NN model focuses on these shapes as they are unique to this texture.

For textures shown in Figure 9 and Figure 10, with more regular patterns, we can see the model focus on more area to recognize the repeated pattern and was able to differentiate between the different repeated patterns correctly.

Additionally, the model does not only focus on the pattern and the shape; it also can recognize the different colors by using the spectral bands in the raw SFA images. In the dataset we used, we had the same pattern with different colors, and the model was robust to notice the difference, so it was clearly able to differentiate between the colors of the textures.

This visualization showed us the ability of the NN SFA model to extract unique features from different textures and was able to adapt the model to different textures. Even though the amount of data we used during training was very little compared to what is usually necessary to train a CNN architecture, the model was able to learn and extract different features from the textures, such as shapes and colors, which allowed the model to robustly perform the classification task. The model also showed reasonably good scalability for different textures and environmental conditions with the addition of extra data with more varieties. The generalization to exposure time was good enough, while the generalization to different illumination was fairly limited and would require more training data. However, this is not the case for the LBP model, as the features the model extracted are quite static and rely strongly on stable magnitudes. Our results showed that CNN could be used for spectral texture classification on the raw SFA data directly without the need for demosaicing. Additional work to compare the NN performance on the raw SFA images and the demosaiced images is required. This will help us determine whether demosaicing will be beneficial for the texture classification task under different environmental conditions. In addition, the drop in performance related to the strong impact of the illumination may be solved by applying spectral constancy [30] to handle the variation in illumination.

## 5. Conclusions

This article focuses on texture classification directly applied to raw Spectral Filter Array images without a preliminary step of demosaicing. We proposed a method for SFA texture classification based on Convolutional Neural Networks. This CNN is pre-trained on ImageNet, then tuned on spectral data. The model performance was compared with the state-of-the-art methods for SFA texture classification on raw images. Differently from the other works that simulate the SFA data from hyper-spectral data, we used a dataset that was captured with an SFA sensor. This dataset allows us to evaluate the model’s performance in real environmental conditions.

Additionally, we investigated the impact of exposure and illumination on the performance of different methods. Our experiment showed the strong effect of different lighting conditions on changing the features extracted from the texture SFA raw image. Our model performance was better in the majority of the cases and was better for adapting to changes in illumination and intensity by increasing the variety in the training data. All the tested models struggled to recognize textures under different illumination than the training setup, but our model showed a better ability to adapt to the addition of data and would probably adapt very well with an increase in data with illumination varieties. Even though the dataset was fairly small compared to what is usually needed, the CNN-based method performance was still better, which shows the ability of CNN architecture to recognize patterns and extract features.

Our work shows once again that illumination is a key factor in imaging. A future direction for this work would be to see how the performance would vary if we embed the concept of spectral constancy in the architecture.

## Figures and Tables

**Table 1:** OBLBP Algorithm Performance on HyTexiLa Dataset.

Experiment	F1-Score
All Textures	0.98
Textile Textures	1

**Table 2:** F1-score for the two methods on SFA textures captured under daylight illumination and 50 ms of exposure time (Daylight–50 ms–training and testing).

Experiment	F1-Score	Precision	Recall
LBP SFA	0.9394	0.9465	0.9396
NN SFA	0.9699	0.9737	0.9704

**Table 3:** F1-score of the different models with all lighting conditions included during training and testing (All Lighting Conditions–50 ms–training and testing). In both models, the recall was more affected than the precision, which shows that the models are predicting more false negatives.

Experiment	F1-Score	Precision	Recall
LBP SFA	0.8273	0.8555	0.8175
NN SFA	0.8684	0.8945	0.8623

**Table 4:** F1-score of the different models with one lighting condition for training (horizon) and another one for testing (daylight). (Daylight for testing–horizon for training–50 ms).

Experiment	F1-Score	Precision	Recall
LBP SFA	0.0519	0.0653	0.0511
NN SFA	0.1582	0.2046	0.1889

**Table 5:** F1-score of the different models with one lighting condition for testing (daylight) and the rest of the lighting conditions for training. (Daylight for testing - Rest of Lighting training–50 ms).

Experiment	F1-Score	Precision	Recall
LBP SFA	0.0884	0.1089	0.1212
NN SFA	0.3312	0.3553	0.3514

**Table 6:** F1-score of the different models with all exposure times included during training and testing (All Exposure times–Daylight–training and testing).

Experiment	F1-Score	Precision	Recall
LBP SFA	0.9524	0.9537	0.9527
NN SFA	0.9684	0.9700	0.9684

**Table 7:** F1-score of the different models with one exposure time for training (20 ms) and another one for testing (50 ms) (50 ms for testing–20 ms for training–Daylight).

Experiment	F1-Score	Precision	Recall
LBP SFA	0.5964	0.5826	0.6431
NN SFA	0.0855	0.0884	0.1188

**Table 8:** F1-score of the different models with one exposure time for testing (50 ms) and the rest of the lighting conditions for training (50 ms for testing–Rest of Exposure times training–50 ms).

Experiment	F1-Score	Precision	Recall
LBP SFA	0.9773	0.9794	0.9779
NN SFA	0.9787	0.9820	0.9791

## Data Availability

Raw SFA images will be made available. The hyperspectral images are available from the HyTexiLa dataset.

## Supplementary Materials

The following supporting information can be downloaded at: www.ntnu.edu/colourlab/software (accessed on 8 June 2023) under the name CID:SFA-Hytexila.
